# Supplementary material for: Identification of Tools for the Functional and Subjective Assessment of Patients in an Aquatic Environment: A Systematic Review
Source: Int J Environ Res Public Health. 2020 Aug 6;17(16):5690. doi: 10.3390/ijerph17165690 (PMC7460483; doi:10.3390/ijerph17165690)
Supplement: Supplementary file 1 [file ijerph-17-05690-s001.pdf]

International Journal of
Environmental Research and Public Health

MDPI

Supplementary Materials: Identification of Tools for the Functional and Subjective Assessment of Patients in an Aquatic Environment: A Systematic Review.

Antonio Cuesta-Vargas [1,2], Jaime Martin-Martin [2,3,*], Manuel Gonzalez-Sanchez [1,2], Jose Antonio Merchan-Baeza [2,4] and David Pérez-Cruzado [2,5]

Table S1. Internal validity analysis (Stard Checklist).

Stand checklist	Cuesta-Vargas et al. 2013 [28]	Cuesta-Vargas et al. 2013 [29]	Cuesta-Vargas et al. 2011 [15]	Nagle et al. 2016 [25]	Gauda et al. 2010 [16]	Louder et al. 2017 [31]	Colado et al. 2009 [30]	Matsumoto et al. 2017 [26]	Matsumoto et al. 2012 [18]	Matsumoto et al. 2013 [20]	Matsumoto et al. 2008 [19]	Chevutschi et al. 2007 [17]	Alberton et al. 2010. [21]	Castillo-Lozano et al. 2014 [27]
1	-	-	-	●	●	●	-	-	-	-	-	-	-	-
2	●	●	●	●	●	●	●	●	●	●	●	●	●	●
3	●	●	●	●	●	●	●	●	●	●	●	●	●	●
4	●	●	●	●	●	●	●	●	●	●	●	●	●	●
5	●	●	●	●	●	●	●	●	●	●	●	●	●	●
6	●	●	●	●	●	●	●	●	●	●	●	●	●	●
7	●	●	●	●	●	●	●	●	●	●	●	●	●	●
8	-	-	-	-	-	-	-	-	-	-	-	-	-	-
9	-	-	●	●	●	●	●	●	●	●	●	●	-	●
10_a	●	●	●	-	●	●	●	●	●	●	●	●	●	●
10_b	●	●	●	-	●	●	●	●	●	●	●	●	●	●
11	●	●	-	-	●	-	-	-	●	●	-	●	●	-
12_a	-	●	-	●	-	-	-	-	-	●	-	●	●	-
12_b	-	-	-	●	-	-	-	-	-	●	-	-	●	-
13_a	-	-	-	●	-		-	-	-	●	-	-	●	-
13_b	-	-	●	-	-	-	-	-	-	-	-	-	-	-
14	●	●	●	-	-	-	-	-	-	-	-	●	-	●
15	-	-	-	-	-	-	-	-	-	-	-	-	-	-
16	●	-	●	●	●	●	●	●	●	●	-	-	●	●
17	●	-	●	●	●	●	●	●	●	●	-	-	●	●
18	-	-	●	-	-	●	●	-	-	-	-	-	●	-
19	-	-	-	-	-	-	-	-	-	-	-	-	-	-
20	●	●	-	●	●	●	-	●	●	●	●	●	●	●
21_a	-	-	-	-	●	-	-	-	-	-	-	-	●	-
21_b	-	-	-	-	-	-	-	-	-	-	-	-	●	●
22	-	-	-	-	●	●	●	●	●	●	-	●	●	●
23	-	-	-	-	-	-	●	-	-	-	-	-	-	-
24	●	●	●	-	-	-	-	-	-	-	-	-	-	-
25	-	-	-	-	-	-	●	-	-	-	-	-	-	-

**26**	●	●	●	●	-	-	-	●	●	-	-	-	-	●
**27**	●	●	-	●	●	●	●	●	●	●	-	●	●	●
**28**	-	-	-	-	-	-	-	-	-	-	-	-	-	-
**29**	-	-	-	-	-	-	-	-	-	-	-	-	-	-
**30**	-	●	-	●	●	-	-	●	-	-	-	●	-	●
**TOTAL**	**15**	**16**	**16**	**15**	**17**	**15**	**15**	**15**	**15**	**16**	**10**	**16**	**17**	**17**

Table S2. Characteristics of included studies.

**Article**	**Task**	**Environments**	**Sample Size (*n*)**	**Variable**	**Criteria Validity**	**Reliability or Measurement Error (ICC)**
Alberton et al. 2011 [21]	Walking	Water and dry	Water: *n* = 14<br>Land: *n* = 11	Cardiorespiratory response	Significant differences between environments and cadence ($p < 0.001$)	Neuromuscular responses<br>RF = 0.942<br>VL = 0.920<br>SM = 0.819<br>BF= 0.764
				Neuromuscular response	No differences between dry and land ($p > 0.100$)<br>Differences at maximal effort ($p = 0.003$)	
				Kinematic response	Significant differences between environments and cadence ($p < 0.001$)	
Chevutschi et al. 2007 [17]	Walking	Water and dry	*n* = 7	Neuromuscular responses	Significant differences in ES and S between environments<br>No significant differences in RF between environments	
Masumoto et al. 2012 [18]	Walking	Water current, walking in water without a water current and walking on dry land	*n* = 7	Cardiorespiratory responses<br>Physiological responses	There was no significant difference in VO_2, RER, HR and VE among the 3 conditions<br>The VO_2, RER, HR, VE, RPE-Br and RPE-Legs obtained while walking in water with a current were significantly higher than those obtained while walking in water without a current, at all speeds<br>There was no significant differences in the SBP and DBP obtained at rest and before exercise among the 3 conditions ($p > 0.05$)	
Masumoto et al. 2008 [19]	Walking at 3 different speeds. Water (1,2; 1,8; 2,4 Km/h) Dry (2,4; 3,6; 4,8 Km/h)	Water and dry	*n* = 9	Neuromuscular responses	The %MVCs obtained from the muscles tested while walking in water were all significantly lower than when walking on dry land at all speed cond itions<br>In contrast, the %MVCs from the VM, RF, BF and GA while walking in water were significantly higher than when walking on dry land at the same speeds	
				Cardiorespiratory responses	There was no significant difference in the VO_2 and HR between environments at moderate and fast speeds, however, in walking speed there VO_2 and HR were significantly higher	
				Rating of perceived exertion	There was no significant differences	

Masumoto et al. 2013 [20]	Walking	Water and dry	$n = 8$	Neuromuscular responses	There was no differences in muscle activity between environments	
Masumoto et al. 2017 [26]	Deep Water Running	Water and dry	$n = 11$	Physiological responses	VO2max and HRmax during DWR were significantly lower than on dry.<br>Significant differences in muscle activity at submaximal and maximal efforts and intensity.	
				Neuromuscular responses	Muscle activity from RF, BF, TA and GA during DWR were significantly lower than that of TMR, regardless of exercise intensity	
				Rating of perceived exertion	There were no significant differences	
Cuesta-Vargas et al. 2011 [15]	Deep water running and cycle ergometry	Water and dry	$n = 23$	Maximal and submaximal physiological responses	Mean maximal and submaximal HRs were significantly lower in the DWR test.<br>No significant differences in blood lactate	
Gayda et al. 2010 [16]	Deep water running in three different protocols: a) <8 min, b) 8–12 min, c) >12 min	Water and dry	$n = 24$	Cardiorrespiratory responses	Maximum HR was significantly lower during the long protocol<br>Maximal running cadencies were lower on the long protocol	Short Deep Water Running protocol:<br>$VO_2 = 0.90$<br>$VCO_2 = 0.92$<br>$VO_2 = 0.84$<br>VE = 0.89<br>RER = 0.65<br>HR = 0.70
	Treadmill				Main peak cardiopulmonary responses obtained during treadmill were significantly higher compared with those obtained on the DWR	
Nagle et al. 2016 [25]	Deep water running	Water and dry	$n = 23$	Cardiorrespiratory responses	A moderate correlation was found between DWR and LTM (land-based treadmill) for VO_2peak ($r = 0.60$; $p < 0.01$), HRpeak ($r = 0.58$; $p < 0.01$) and O_{2pulse} ($r = 0.63$, $p < 0.01$)	VO_2peak = 0.73<br>HRpeak = 0.82<br>O_2pulse = 0.77
Castillo-Lozano et al. 2014 [27]	Shoulder scaption	Water and dry (different planes and speed)	$n = 16$	Neuromuscular response	Significant differences in:<br>During flexion, the *p* and MD at 90/s and 45/s<br>During abduction, the P and LD at the 3 Speeds (30/s, 45/s and 90/s)<br>During scaption, the P and MD muscle at the 3 speeds (30/s, 45/s and 90/s)	
Cuesta-Vargas et al. 2013 [28]	Sit to stand	Water and dry	$n = 10$	Neuromuscular responses	The %MVC contraction was different for all muscles during between environments	
Cuesta-Vargas et al. 2013 [29]	Time Get up and go	Water and dry	$n = 10$	Neuromuscular responses	The %MVC was significantly different ($p < 0.05$) for majority of the muscles tested during the TUG (RF, BF, TA, S, GA and ES)	

Colado et al. 2009 [30]	Jump	Water and dry	*n* = 12	Kinematic responses	Maximum concentric force was greater in water<br>Peak impact force was lower for the aquatic jumps
Louder et al. 2017 [31]	Jump	Water and dry	*n* = 67	Kinematic responses	Unweighting times were longer and propulsive times were shorter in water versus land<br><br>Unweighting times and percent of time in unweighting were greater in older adults<br>All measures of power, amortization rate and force (BW) were greater in water versus land.
				Kinetic responses	Maximum shank flexion angle, peak shank extension velocity and peak dorsiflexion velocity were greater in water versus land<br>Maximum plantarflexion angle, peak thigh flexion velocity and peak shank flexion velocity were greater on land versus water

BF, Biceps femoris; BW, body weight; DBP, Diastolic blood pressure; DWR, Deep water running; ES, Erector spinae; GA, Gastrocnemius; HR, Heart rate; LT, latissimus dorsi; MD, middle deltoid; MVC, Maximum voluntary contraction; P, Pectoralis; RF, Rectus femoris; RER, respiratory; RPE, rate of perceived exertion; exchange ratio; S, Soleus; SBP, Systolic blood pressure; ST, Semitendinosus; TA, Tibialis anterior; TMR, treadmill running; VE, Ventilation; VL, Vastus lateralis; VM, Vastus medialis.

Table S3. Structural Characteristics of the questionnaires.

Questionnaire	Acronym	Nº Items	Sub-category	Time to Complete	Item Rated	Cutoff	Cost
**Activities-specific Balance Confidence Scale [19,36,39,42,43-52]**	ABC scale	16	Balance confidence	5–10 min	0–100	Scores <67% indicates a risk for falling	Not specified
**Arthritis Impact Measurement Scale [53-56]**	AIMS2	78	Mobility Level<br>Walking and Bending<br>Hand and Finger Function<br>Arm Function<br>Self- Care Tasks<br>Household Tasks<br>Social Activity<br>Support from Family and Friends<br>Arthritis Pain<br>Work<br>Level of Tension<br>Mood<br>Satisfaction with health area	20 min	Yes/No<br>4–5 and 6 point Likert scales	Not specified	Free
**Assessment of Motor and Process Skills [57-63]**	AMPS	16 motor skills<br>20 process skills	Motor skills:<br>• Body position<br>• Obtaining and holding objects<br>• Moving self and objects<br>• Sustaining performance<br>Process skills:<br>• Sustaining performance<br>• Applying knowledge<br>• Temporal organizarion<br>• Organizing Space and Objects<br>• Adapting Performance	30–40 min	1–6	Scores below 2.0 logit for process scale indicate increased need for assistance to live in the community<br>Scores below 1.0 logit for motor scale indicate increased need for assistance to live in the community [2]	795$
**Assessment of Quality of Life Scale; [39,64,65]**	AQoL	15	Illness<br>Independent living<br>Social relationships<br>Physical senses<br>Psychological wellbeing	5 min	A-D	-	Free
**Arthritis Self-Efficacy Scale [65]**	ASES	20	Pain<br>Function<br>Other symptoms	5 min	1–10	-	-
**Berg Balance Scale [34,36,66-70]**	BBS	14	Balance and functional mobility	15–20 min	0–4	Score of 56 indicates functional balance<br><br>Score of <45 indicates individuals may be at greater risk of falling [3]	Free
**Child Health Assessment Questionnaire [71,72]**	CHAQ	16	Family cohesion<br>Global health<br>Physical functioning<br>Self-esteem	30–40 min	Yes/No items<br><br>Multiple answers	Not specified	Free

**Chronic Venous Insufficiency Questionnaire [73-75]**	CIVIQ2	4	Psychological functioning<br>Physical functioning<br>Social functioning<br>Pain	10 min	1–5	Not specified	Free
**Client Satisfaction Questionnaire [76-78]**	CSQ	3,4,8,18 and 31 versions	None	10 min	1–4	Not specified	Free
**Disease Activity Score [79-82]**	DAS	21 and 28 versions	Proximal interphalangeal joints<br>Metacarpophalangeal joints<br>Wrists<br>Elbows<br>Shoulders<br>Knees	10 min	0–100	< 2.6 Remission of disease severity<br>≥ 2.6 - < 3.2 Low disease severity<br>≥ 3.2 - ≤ 5.1 Moderate disease severity<br>> 5.1 High disease severity	Free
**EuroQol-5 Dimension questionnaire [83,84]**	EQ-5D	6	Mobility<br>Self-care<br>Usual activities<br>Pain/discomfort<br>Anxiety/depression	<5 min	1)None<br>2)Mild to moderate<br>3)Severe	-	Not specified
**Falls Efficacy Scale [85-88]**	FES-I	16	Activity<br>Participation	10 min	1–4	Scores between 16–19 indicate low concern about falls<br>Scores between 20–27 indicate moderate concern about falls<br>Scores betwenn 28–64 indicate nigh concern about falls[4]	Not specified
**Fibromyalgia Impact Questionnaire [89-92]**	FIQ	10	-	10 min	Different rates:<br>0–3<br>0–7<br>0–5<br>Visual Analogue Scale	-	Free
**Generalized Anxiety Disorder Scale – 7 [92]**	GAD-7	7	Anxiety symptom levels	≥5 min	0–21	Anxiety symptom levels<br>0–4: minimal<br>5–9: mild<br>10–14: moderete<br>15–21: severe	Free
**Health Assessment Questionnaire-modified [93-95]**	HAQ	41 questions<br>20: 0–3 Liknkel scale<br>13 + 8 dicotomic	Vestirse y arreglarse/Dressing and grooming,<br>Levantarse/Arising,<br>Comer/Eating,<br>Caminar/Walking,<br>Higiene/Hygiene,<br>Alcanzar/Reach,<br>Agarrar/Grip,<br>Actividades/Activities	5–10 min	0–60	-	Free
**Knee Injury and Osteoarthritis Outcome Score Questionnaire [96-99]**	KOOS:	42 questions	Knee-related quality of life<br>Activities of daily living	10 min	0–100	Pain: <86.1<br>Symptoms: <85.7	free

			Sport and recreation function<br>Symptoms<br>Pain			ADL: <86.8<br>Sport/Rec: <85.0<br>QoL: <87.5	
**Movement Disorder Society - Unified Parkinson’s Disease Rating Scale [100-102]**	MDS-UPDRS	50	Non-motor experiences of daily living<br>Motor experiences of daily living<br>Motor function<br>Motor fluctuations and dyskinesia	20–30 min	0–200	−3.5 / 4.5	Free
**Physical Activity Scale for the Elderly [103-108]**	PASE:	12	Activities of Daily Living<br>General health	<5min	0–400	-	free
**Parkinson’s Disease Questionnaire [109-112]**	PDQ39:	39	Mobility<br>Activities of daily living<br>Emotional well-being<br>Stigma<br>Social Support<br>Cognition<br>Communication<br>Bodily discomfort	10–20 Min	0–100	-	Free
**Quebec Back Pain Disability [113]**	QBPDS	20	Disbility	<5min	0–100	-	Free
**Short Form-12 [114]**	SF-12	12	2 components: physical components.<br>mental components.<br>8 sub-scales<br>Physical health state<br>Mental health state<br>Physical Functioning<br>Role Limitations fue to Physical Problems<br>Genera health perceptions<br>Vitality<br>Social Functioning<br>Role Limitations due to Emotional Problems<br>General Mental Health	3–5 min	0–100	-	Not Free
**Short Form-36 [114-119]**	SF-36	36	2 components: physical components.<br>mental components.<br>8 sub-scales<br>Physical health state<br>Mental health state<br>Physical Functioning<br>Role Limitations fue to Physical Problems<br>Genera health perceptions<br>Vitality<br>Social Functioning<br>Role Limitations due to Emotional Problems<br>General Mental Health<br>Health Transition	10–45 Min	0–100	-	Not Free

**Tampa Sacale of Kinesiophobia [120]**	TSK-13	13	Kinesiophobia<br>activity avoidance<br>somatic focus	<10 min	0–52	-	Free
**Venous Clinical Severity Score [121]**	VCSS	10	Clinical severity	< 5min	0–30	-	Free
**Western Ontario McMaster Universities Osteoarthritis Index [122-125]**	WOMAC	24	Pain<br>Stiffness<br>Function	10–20 min	0–96	-	Not free

Table S4. Psychometric characteristics of the questionnaires.

Questionnaires	Reliability Test-Retest (Respuesta al Ítem) ICC	Internal consistency (α-Crombach)	Construct Validity	Factor Analysis	Sensitivity / Specificity	SEM	MDC
**Activities-specific Balance Confidence Scale (ABC)**	0.53 [19]–0.96 [36]	0.91 [42]–0.96 [43]	Functional gait assessment:<br>*r* = 0.53 (95% CI) [38]<br>Fear of falling Avoidance Behaviour Questionnaire:<br>*r* = −0.67 (95% CI) [43]<br>Berg Balance Scale:<br>*r* = 0.75 (95% CI) [44]<br>Timed up & go test:<br>*r* = 0.70 (95% CI) [51]	1 Factor	58%–97%/96%–32% [50]	6.81 [5]–1.19 [36]	11.12 [13]-13.00 [48]
**Arthritis Impact Measurement Scale (AIMS)**	0.81–0.94 [53]	0.32 [16]–0.90 [55]	WOMAC<br>*r* = 0.16–0.22 (95% CI) [54]<br>100 mm visual analog scale (VAS) for pain<br>*r* = 0.18–0.41 [95% CI] [54]<br>Range of motion<br>*r* = 0.17–0.44 (95% CI) [54]	13 factors		1.37 [55]	3.80 [56]
**Assessment of Motor and Process Skills (AMPS)**	0.60 [36]–0.95 [57]	0.79 [22]–0.92 [57]	Pain frequency<br>*r* = −0.30 (95% CI) [58]<br>Pain intensity<br>*r* = 0.36 (95% CI) [59]<br>Disease duration<br>*r* = 0.39 (95% CI)	2 factors	67%–81%/70%–72% [63]	1.96 [61]	-
**Assessment of Quality of Life Scale (AQLS)**	0.26–0.78 [39]	0.81 [39]	EuroQol-5D<br>*r* = 0.73–0.76 (95% CI) [65]<br>Health utilities index<br>*r* = 0.79–0.82 (95% CI) [65]<br>SF-6D<br>*r* = 0.77–0.80 (95% CI) [64]<br>Quality of Well-being<br>*r* = 0.65–0.67 (95% CI) [64]	5 factors	-	-	-
**Arthritis Self-Efficacy Scale [ASES)**	0.75–0.92 [65]	0.76–0.89 [65]	Pain frequency<br>*r* = −0.30 [95% CI) [65]<br>Pain intensity	3 factors	-	-	-

			r = 0.36 [95% CI) [45]<br>Disease duration<br>r = 0.39 (95% CI) [45]				
**Berg Balance Scale (BBS)**	0.72 [46]–0.99 [36]	0.86 [48]–0.98 [66]	Two min walk test:<br>r = 0.78(95% CI) (49)<br>10 min walk test:<br>r = 0.79(95% CI) [70]<br>Timed up and go<br>r = −0.82(95% CI) [69]	1 factor	53% [33]–91% [34]/82% [68]–96% [34]	1.49 [29]–2.93 [69]	2.50 [36]–8.10 [70]
**Child Health Assessment Questionnaire (CHAQ)**	-	0.62–0.94 [71]	-	4 factors	SRM: 0.91–1.28 [72]	-	-
**Chronic Venous Insufficiency Questionnaire (CVIQ)**	0.81 [73]–0.98 [73]	0.67 [74]–0.92 [74]	-	4 factors	-	-	-
**Client Satisfaction Questionnaire (CSQ)**	0.41–0.86 [76]	0.83–0.85 [77]	Parent satisfaction questionnaire<br>r = 0.52 (95% CI) [77]<br>Parents'benefit rating<br>r = 0.47 (95% CI) [78]<br>Global assessment of functioning<br>r = 0.37 (95% CI) [78]<br>Therapists´benefit rating<br>r = 0.41 (95% CI) [76]	2 factors	-	-	-
**Disease activity score (DAS)**	0.61–0.91 [79]	0.39 [61]–0.91 [82]	Simplified Ankylosing Spondylitis Disease Activity Score<br>r = 0.83 (95% CI) [80]<br>Ankylosing Spondylitis Disease Activity Score<br>r = 0.81 (95% CI) [81]<br>Patient's pain rating<br>r = 0.59 (95% CI) [81]	1 factor	-	3.01–3.66 [81]	-
**EuroQol-5 Dimension questionnaire (EQ5D)**	-	0.36–0.90 [83]	UK-SF36<br>r = 0.48–0.60 (95% CI) [84]<br>EQ-5D-3L<br>r = 0.74 [95% CI) [84]<br>EQ Visual analogue scale<br>r = 0.48 [95% CI) [83]	5 factors	-	-	-
**Falls Efficacy Scale (FES)**	0.58 [85]–0.96 [87]	0.79 [68]–0.96 [88]	Previous falls<br>r = 0.46(95% CI) [86]<br>Fatigue<br>r = 0.42 (95% CI) [86]<br>Trail making test (Part B)<br>r = 0.28 (95% CI) [86]<br>Muscle strength<br>r = −0.26 (95% CI) [86]<br>Balance measures<br>r = 0.30(95% CI) [86]	1 factor	36% [85]	0.19 [86]–6.4 [85]	0.52 [86]–17.7 [85]

**Fibromyalgia Impact Questionnaire (FIQ)**	0.31 [92]–0.95 [89]	0.80 [91]–0.92 [89]	Health Assessment Questionnaire<br>*r* = 0.25–0.67 (95% CI) [90]<br>Fibromyalgia Health Assessment Questionnaire<br>*r* = 0.25–0.70 (95% CI) [90]<br>Medical Outcomes Survey Short Form36<br>*r* = 0.19–0.62 (95% CI) [90]<br>Symptom Checklist-90-Revised<br>*r* = 0.20–0.68 (95% CI) [90]	1 factor	-	-	-
**Generalized Anxiety Disorder Scale 7 (GAD-7)**	0.83 [92]	0.92 [92]	PHQ-2 depression scale:<br>*r* = 0.64 (95% CI: 0.63–0.66) [92]<br>Rosenberg Self Esteem Scale:<br>*r* = 0.43 (95% CI: 0.41–0.46) [92]<br>Questionnaire on Life Satisfaction:<br>*r* = 0.34 (95% CI: 0.32 to 0.37) [92]<br>Resilience Scale:<br>*r* = 0.29 (95% CI: 0.26 to 0.31) [92]	1 factor	89%/82% [92]	-	-
**Health Assessment Questionnaire-modified (HAQ-M)**	0.87–0.99 [93]	0.90 [94]	VAS pain [95] (0–3) 0.634<br>Depression [95] (0–9.9) 0.491<br>ESR[4] (mm/h) 0.319<br>Duration[4] (years) 0.22	−1 factor [93]	-	-	
**Knee Injury and Osteoarthritis Outcome Score Questionnaire; (KOOS)**	0.73–0.93 [98]	Pain: 0.82 [97]<br>Symptoms:0.78 [97]<br>ADL: 0.79 [97]<br>Sport/Rec: 0.80 [97]<br>QoL: 0.82 [97]	SF-36: 0.03–0.74 [99]	-	-	Pain: 2.2 [98]<br>Symptoms:3.18<br>ADL: 2.9 [98]<br>Sport/Rec: 2.18<br>QoL: 206 [98]	Pain: 6.1<br>Symptoms:8.5 [99]<br>ADL: 8.0 [99]<br>Sport/Rec: 5.8 [96]<br>QoL: 7.2 [97]
**Movement Disorder Society - Unified Parkinson's Disease Rating Scale (MDS-UPDRS)**	ICC ≥ 0.92 - ICC ≥ 0.96 [101]	α ≥ 0.96 [101]	C-reactive protein:<br>*r* = 0.004–0.822.<br>DextQ-24: *r* = 0.50–0.66 [101]<br>Non-Motor Symptoms Scale: *r* = 0.81 [102]<br>TSK-13: *r* = 0.513 [101]	4 factors	0.94 / 0.70 [101]<br>0.78 / 0.95 [101]	-	-
**Physical Activity Scale for the Elderly (PASE)**	0.77 [104]	0.71 [107]	Actigraph GT1M –0.30 [103]<br>CHAMPS: *r* = 0.58–0.64 [103]<br>IPAQ – 0.61 [105]<br>SF-36: *r* = 0.17–0.30 [107]<br>YPAS: *r* = 0.61 [107]<br>6MWT: *r* = 0.68 [107]	-	-	31 [108]	87 [108]
**Parkinson's Disease Questionnaire (PDQ39).**	Test-Retest: 0.68–0.95 [109]<br>Mobility: 0.89–0.95 [109]<br>ADL: 0.93–0.96 [109]	Test-Retest [91]: 0.84–0.94<br>Mobility: 0.85–0.96<br>ADL: 0.83–0.94<br>Emotional well-being: 0.79–0.91	EuroQoL-5d: *r* = 0.75 [92]<br>SF-36: *r* = 0.34–0.80 [92]<br>Beck's DI: 0.73 [93]	-	-	Mobility: 6.25<br>ADL: 8.54<br>Emotional well-being: 7.26	Mobility: 12.24<br>ADL: 16.72<br>Emotional well-being: 14.22<br>Stigma: 21.21

	Emotional well-being: 0.90–0.95 [109]<br>Stigma: 0.88–0.95 [109]<br>Social Support: 0.66–0.92 [109]<br>Cognition: 0.84–0.93 [109]<br>Communication: 0.86–0.90 [109]<br>Bodily discomfort: 0.80–0.91 [109]	Stigma: 0.54–0.90<br>Social Support: 0.13–0.87<br>Cognition: 0.6–0.87<br>Communication: 0.65–0.87<br>Bodily discomfort: 0.56–0.87				Stigma: 10.82<br>Social Support: 12.50<br>Cognition: 11.29<br>Communication: 10.74<br>Bodily discomfort: 12.49 [112]	Social Support: 24.50<br>Cognition: 22.12<br>Communication:<br>Bodily discomfort: 24.48 [112]
**Quebec Back Pain Disability (QBPDS-PT)**	0.86–0.99 [113]	0.895–0.96 [113]	RMDQ: 0.60–0.70 [113]<br>ODI: 0.68–0.81 [113]<br>VAS: 0.37–0.87 [113]<br>SF-36: 0.64–0.69 [113]	1–7 factors	-	-	11.04–32.9 [113]
**Short-Form 12 (SF-12)**	0.60–0.78 [114]	0.82–0.88 [114]	EuroQoL: r = 0.38–0.61 [114]	8 Factors	70% [114]	-	3.77 [114]
**Short-Form 36 (SF-36)**	0.71–0.89 [114]<br>0.72–0.87 [115]	0.74–0.93 [115]<br>0.76–0.98 [116]	QOL-DAv2.0: 0.42–0.75 [118]<br>NMS: 0.40–0.52 [118]<br>WHO-DAS II: 0.52–0.70 [119]<br>EuroQoL: 0.66 [119]<br>Barthel Index: 0.217–0.810 [118]	8 Factors	-	1.2–3.5 [117]	19–45 [118]
**Tampa Scale of Kinesiophobia. (TSK-13)**	0.90 [120]	0.94 [120]	PCS: *r* = 0.54 [120]<br>FES: *r* = 0.710 [120]<br>TSK-13: *r* = 513 [120]<br>HADS: *r* = 0.443–0.626 [120]<br>SF-36: *r*= 0.236–0.563 [120]	2 factors	-	-	10.7 [120]
**Venous Clinical Severity Score; (VCSS)**	0.92 [121]	κ = 0.68 [121]	CIVIQ: *r* = 0.30–0.55 [121]<br>CEAP: *r* = 0.21–0.51 [121]	-	-	-	-
**Western Ontario McMaster Universities Osteoarthritis Index (WOMAC)**	0.77 [124]–0.98 [123]	0.84 [124]–0.98 [122]	SF-36: *r* = 0.67–0.73 [125]<br>COAT-Scale: *r* = 0.92 [124]	-	-	124	3.94 [104]–15.3 [123]

© 2020 by the authors. Licensee MDPI, Basel, Switzerland. This article is an open access article distributed under the terms and conditions of the Creative Commons Attribution (CC BY) license (http://creativecommons.org/licenses/by/4.0/).
